# Frequency and Risk Factors Associated with Prematurity: A Cohort Study in a Neonatal Intensive Care Unit

**DOI:** 10.3390/jcm13154437

**Published:** 2024-07-29

**Authors:** Maria Goretti Policarpo Barreto, Maria Conceição Manso, Renata Policarpo Barreto, Roberta Policarpo Barreto, Lara Moreira Teles de Vasconcelos, Cláudia Silva

**Affiliations:** 1 Faculdade de Ciências e Tecnologias, Universidade Fernando Pessoa, Praça 9 de Abril 349, 4249-004 Porto, Portugal; 2 Hospital Regional Unimed Fortaleza (HRU), Avenida Visconde do Rio Branco, 400, São João do Tauape, Fortaleza 60420-570, Brazil; rpolicarpobarreto@gmail.com (R.P.B.); isis_roberta@yahoo.com.br (R.P.B.); laramtvasconcelos@gmail.com (L.M.T.d.V.); 3 Faculdade de Ciências da Saúde, RISE-Health, Universidade Fernando Pessoa, Rua Carlos da Maia, 296, 4200-150 Porto, Portugal; csilva@ufp.edu.pt; 4 FP-I3ID, FP-BHS, Universidade Fernando Pessoa, Praça de 9 de Abril 349, 4249-004 Porto, Portugal; 5 REQUIMTE-LAQV (Laboratório Associado de Química Verde), Universidade do Porto, 4050-313 Porto, Portugal; 6 Centro de Ciências da Saúde, Universidade de Fortaleza (UNIFOR), Avenida Washington Soares, 1321, Edson Queiroz, Fortaleza 60811-905, Brazil; 7 Centro de Ciências da Saúde, Universidade Federal do Ceará (UFC), Avenida da Universidade, 2853, Benfica, Fortaleza 60020-181, Brazil

**Keywords:** maternal–fetal risk factors, neonatal mortality, potable water, premature labor, prematurity, prevalence, sewage system available

## Abstract

**Background/Objectives**: Prematurity rates remain high and represent a challenge for the public health systems of any country, with a high impact on neonatal mortality. This study aimed to evaluate the frequency and environmental and maternal–fetal risk factors for premature birth in a cohort of parturient women, with their newborns monitored in a neonatal intensive care unit at a private reference hospital. **Methods**: A cohort was carried out between 2013 and 2018 among parturient women living in a capital city in the Northeast of Brazil whose newborns were admitted to the neonatal intensive care unit. This study was approved by the Research Ethics Committee of the University of Fortaleza. The information collected comprised data from both medical records and hydrosanitary data from maternal homes. **Results**: The prevalence of prematurity among live births (*n* = 9778) between 2013 and 2018 at this hospital was 23%. The frequency of prematurity among those eligible (*n* = 480) was 76.9%, and the frequency of eligible premature babies (*n* = 369) in relation to the total number of births in this period was 3.8%. In the multivariate analysis, the significant risk factors for prematurity were primigravida (RR = 1.104, 95%CI: 1.004–1.213) and hypertensive syndromes during pregnancy (RR = 1.262, 95%CI: 1.161–1.371), and the significant protective factor was the highest number of prenatal consultations (RR = 0.924, 95%CI: 0.901–0.947). **Conclusions**: This study contributes to providing greater visibility to prenatal care and the understanding of complications during pregnancy and childbirth care. These results indicate the need to implement public policies that promote improvements in the population’s living conditions and care for pregnant women to reduce premature births and, consequently, neonatal and infant mortality.

## 1. Introduction

Preterm births are those occurring before 37 full weeks of gestation [1,2] or less than 259 days [3], counted from the first day of the mother’s last menstrual period [1,2,3], and can occur spontaneously or electively, when there is clinical indication due to complications related to the pregnant woman and/or the fetus [2,4].

In 2010, the estimated global prevalence was 11.1% (95% CI: 9.1% and 13.4%) in 99 countries [5]. Around 15 million preterm births occurred worldwide, equivalent to more than one preterm birth in ten in 2015 [6]. That same year, in the United States, rates of premature births (<37 weeks of GA) were 9.6% of total births. Thus, in 2021, the preterm birth rate rose for the seventh consecutive year to 10.5% of total births in the United States [7]. Therefore, prematurity rates remained high and constituted a challenge for the public health systems and economics of any country [6].

Furthermore, in Brazil, there were 2,849,146 live births in 2019, of which 315,831 (11%) were preterm, and 43,233 had a gestational age (GA) between 22 and 31 weeks. The spatial distribution of these preterm births varied between regions. The Northeast region ranked second in absolute numbers of preterm births, accounting for 86,601 preterm births in 2019, while the Midwest region had the lowest number, 26,545 preterm births. The state of Ceará recorded 129,185 live births, of which 14,973 were preterm, and the city of Fortaleza had a proportion like the state and national averages, with 11.5% preterm infants with GA <37 weeks (10,913 preterm births) [8]. Global data from the World Health Organization (WHO) disclosed that, in that same year, Brazil ranked 10th in the world in absolute number of preterm births, which is a matter of concern for the country’s public health scenario [9].

In the world, there were 55.4 million deaths in general in 2019, with 55% of these deaths corresponding to the ten main causes identified by global health estimates. Preterm birth complications, added to other neonatal conditions, ranked fifth in this list, causing 2 million deaths [9].

Prematurity is considered the main cause of infant mortality in children under five years of age [10,11,12,13]. In addition, most of these deaths could be prevented with low-cost interventions in the prenatal period [2,10].

In recent years, preterm newborns have shown greater survival. Surviving preterm infants are at greater risk for several short- and long-term morbidities, which can be minimized with early multidisciplinary interventions [14], such as delayed neuropsychomotor development [15,16], learning difficulties, and visual and hearing impairment [17].

Existing studies have shown that the etiology of preterm births is not fully understood. Several studies have considered it a multifactorial, complex event [12,18,19], varying between countries and regions [2,20]. Some risk factors for preterm birth are reversible, and others are not [21]. Epidemiological studies have identified several socioeconomic, demographic, genetic, and health factors as causes of preterm births, namely maternal age extremes (under 19 years of age [22,23,24] and over 34 years of age [21,22,25,26,27]) and low level of education [23]. Gynecological, obstetric, and clinical causes during pregnancy have been researched, such as assisted reproduction in current pregnancy [21], primiparity [12], previous prematurity’s history [18,21,28], past history of procedural abortion [21] and stillbirth [24], short or dilated cervix in the mid-trimester (16 to 28 weeks of gestation) [21], multiple pregnancy [2,18,19,21,24,28,29,30,31], pregnancy-specific hypertensive syndrome [12,13,24,26,27,29,30], urinary tract infection [13,28], and antepartum the ovular membranes’ rupture [21,26,32]. Absent, inadequate, or lack of prenatal care (absence of or <5 prenatal consultations) [12,21], cesarean section [22,26,27,30], pre-existing diabetes [2,24] or gestational [24,30], chronic arterial hypertension [2,13,25], and low Apgar scores in the first [19] and fifth minutes of life [19,25] have been identified as risk factors for preterm birth.

Furthermore, several studies have described an association between preterm labor/birth and a lot of genital tract infections/colonization [21,33,34,35,36,37,38].

Regarding fetal and infant risk factors, existing studies have shown that being male, certain congenital anomalies (chromosomal trisomy’s 21, 18, and 13), growth restriction, alloimmunization, and hydrops fetalis are risk factors for spontaneous preterm birth [21].

Considering the potential effect of prematurity on the increase in perinatal, neonatal, and infant mortality rates in the country, especially in the North and Northeast regions, and numerous morbidities, it was essential to identify the risk factors associated with preterm birth, aiming to implement early interventions to prevent or control them, minimizing their impact on the outcome.

The present study aimed to determine preterm birth frequency and identify the environmental, maternal, and fetal factors associated with prematurity among live births admitted to the neonatal intensive care unit (NICU) of a referral hospital. The importance of this study is related to the fact that prematurity is one of the main determinants of neonatal and postneonatal morbimortality in several studies carried out in Brazil.

## 2. Materials and Methods

This study was carried out in a cohort of parturients and their newborns, conducted over a period of 6 years between January 2013 and December 2018 in a private hospital of the supplementary health network in Fortaleza, state of Ceará, Brazil. The sample consisted of all newborns admitted to the NICU soon after delivery, as well as their mothers. All deliveries occurred in the obstetric center of this institution, which is a state and national referral hospital for the care of high-risk pregnancies.

The following exclusion criteria were utilized: being a resident of another municipality, having multiple births, having severe malformations incompatible with life, or being transferred to another institution.

The dependent variable (outcome) was preterm birth. According to the GA, preterm newborns were classified as extremely preterm (less than 28 weeks), very preterm (28 completed weeks to 31 weeks and six days), moderately preterm (32 to 33 weeks and six days), and late preterm (34 to 36 weeks and six days) [1,2,3]. Newborns with a GA of 37 to 41 weeks and six days were called full-term. GA was determined using the best estimate from early ultrasound (when performed up to 20 weeks of gestation), the date of the last menstrual period, or the newborn’s physical examination (Capurro or New Ballard method), which was used when there was a discrepancy between the last two methods [39].

The evaluated independent variables were environmental, maternal, and newborn factors. The environmental factors included the hydrosanitary conditions of the mother’s home (access to clean water, sewer network connected to the home); the maternal factors included sociodemographic, gestational, and childbirth characteristics. The sociodemographic characteristics were age (>35 and ≤35 years), marital status, and level of education. The variables related to pregnancy/delivery included prenatal consultations, delivery type (vaginal or cesarean section), number of pregnancies, parity, history of miscarriage, clinical/obstetrical complications during pregnancy [hypertensive syndrome, previous or gestational diabetes mellitus, thrombophilia, infectious diseases (urinary infection and TORCHS—toxoplasmosis, rubella, cytomegalovirus, herpes simplex and syphilis), premature placental abruption, placenta previa] and presence of complications during labor. Given the low frequency of some variables, it was necessary to group them together when analyzing the data, as was the case with hypertensive syndrome during pregnancy, which includes mild pre-eclampsia, severe pre-eclampsia, eclampsia, and HELLP syndrome. Prenatal care was considered inadequate for records of fewer than five consultations during pregnancy, regardless of GA.

Regarding the newborn’s variables, sex, presentation at delivery (cephalic and pelvic/cormic) and nutritional status were evaluated. Nutritional status was assessed using percentiles of intrauterine growth curves. Birth weight was classified as small for GA (when below the 10th percentile), adequate for GA (between the 10th and 90th percentiles), and large for GA (above the 90th percentile) [40]. The WHO intrauterine growth percentile curves were adopted. When analyzing the data, it was decided to group the two upper classes together (adequate for GA and large for GA).

Data on the characteristics of postpartum women and their newborns were collected using their respective medical records. Data related to the sewer network and water supply were obtained from the Water and Sewer Company of Ceará based on the maternal household address.

The statistical analysis of the data was performed using IBM SPSS Statistics 25^®^ (IBM Corp., Armonk, NY, USA, 2019). Independent variables were described using point estimates after being grouped into sociodemographic and environmental maternal characteristics, conditions of pregnancy and childbirth/maternal complications, and characteristics of newborns.

To verify which of these variables, in multiple contexts, significantly influenced prematurity, they were analyzed in blocks, considering a 5% significance level and a 95% confidence interval. To explore the relationships between the variables, bivariate analysis of the variables categorized in contingency tables was performed using Pearson’s chi-square test or Fisher’s exact test. The bivariate analysis of quantitative variables was performed using the Mann–Whitney test. Poisson regression with robust variation and Wald statistics were used in the multivariate model construction. As a measure of association between factors of interest and prematurity, point estimates of relative risk (RR) and 95% confidence intervals were calculated. In the multiple contexts, variables whose bivariate analysis showed an association with significant prematurity up to a significance level of 15% (*p* = 0.15) were introduced. The previously selected variables were submitted to a new multivariate analysis using factors with a significance level of 5% as parameters for permanence in the final model.

This study complied with the Regulatory Guidelines and Norms for Research Involving Human Beings of the National Health Council (CNS Resolution 466/2012), having been submitted to the Research Ethics Committee of the University of Fortaleza—UNIFOR (protocol code 137/15) and to “Plataforma Brasil” and approved by the Research Ethics Committee of UNIFOR in 2016 (CAAE number: 51625815.7.0000.5052, opinion number 1.602.450, at 20 June 2016). The risks inherent to this research were minimal since the analysis of medical records was carried out, and the privacy and anonymity of the collected information were ensured.

## 3. Results

**Figure 1:**
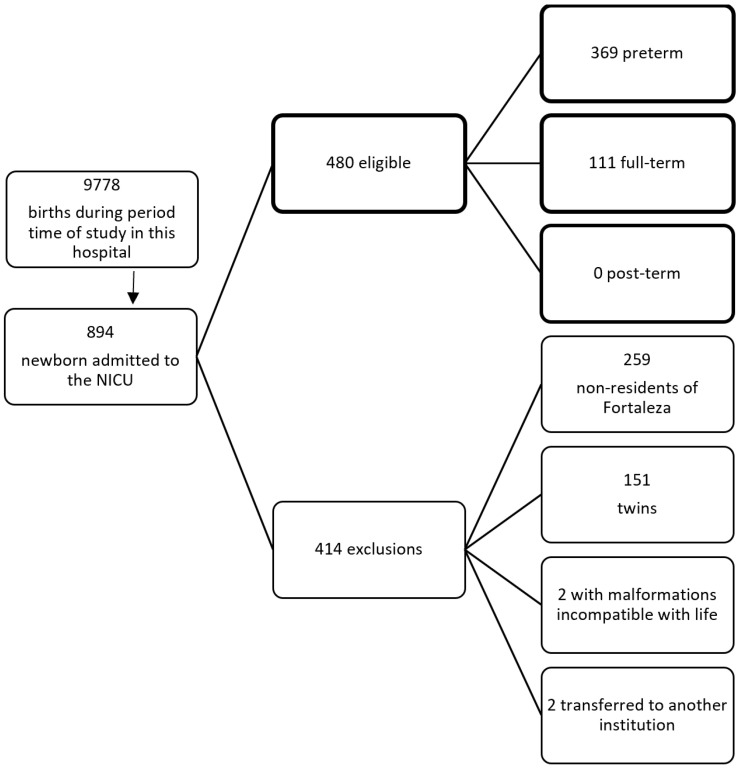
Flowchart describing the births during the study period, those admitted to the NICU, and those excluded from this study according to the previously defined criteria.

During the study period, 9778 children were born alive in this hospital. Of these, 894 (9.1%) were admitted to the NICU, making up the total study population. According to the previously established criteria, 414 neonates (46.3%) were excluded (259 because their mothers did not live in Fortaleza, 151 because they were the result of multiple pregnancies, two because they presented with severe malformations incompatible with life, and two because they had been transferred to another institution) Figure 1.

The prevalence of prematurity among live births (*n* = 9778) between 2013 and 2018 at this hospital was 23%. The frequency of prematurity among those eligible (*n* = 480) was 76.9%, and the frequency of eligible premature births (*n* = 369) in relation to the total number of births in this period was 3.8%. Of our NICU sample of 480 parturients, 369 had preterm births, of which 196 (53.1%) had a GA of less than 34 weeks [41 (11.1%) were extremely preterm (22 to 27 weeks and six days), 63 (17.1%) were very preterm (28 to 31 weeks and six days), and 92 (24.9%) were moderately preterm (32 to 33 weeks and six days), and less than half of these preterm births; that is, 173 (46.9%), were late preterm births (34 to 36 weeks and six days)].

Table 1 shows the characteristics of parturients and newborns and the analysis of association and RR estimates of these variables for the outcome of prematurity.

As for the parturients, it was found that their age ranged from 13 to 58 years (mean of 31 ± 5.7 years), only 21.9% were older than 35 years, and most were married or had a common-law marriage (75.6%) and had a higher frequency of complete higher education (65.2%). Most maternal households (51.5%) had access to sewer systems, and almost all (98.1%) had access to clean water. When comparing the group of preterm infants with the full-term ones, no significant differences were found regarding these maternal characteristics or environmental factors.

The number of prenatal consultations ranged from 1 to 15, with the preterm group having a significantly lower average than the full-term newborns (6 ± 2.2 versus 8 ± 2.2). Only 13.5% (*n* = 65) of all parturients had fewer than five prenatal consultations, of which 93.8% (*n* = 61) were mothers of preterm newborns and 6.2% (*n* = 4) of full-term newborns.

In this sample, the number of pregnancies ranged between one and eight. Among the primigravidae, prematurity was significantly more frequent (57.5% versus 44.1%). The previous history of miscarriages in the sample was unusual (22.1%), and no differences were found regarding this variable between the two newborn groups. As for clinical/obstetric complications, it was observed that only hypertensive syndrome (including mild pre-eclampsia, severe pre-eclampsia, eclampsia, and HELLP syndrome) was significantly more common in the preterm group (30.6% versus 9.9%). In the total sample, 7.9% of the mothers had gestational diabetes (all were controlled exclusively with dietary advice, none required insulin, and none had previous diabetes mellitus), 17.9% had a urinary infection, 2.7% had TORCHS, 2.9% had thrombophilia, and 6.3% had bleeding in the third trimester of pregnancy. No significant differences were found for any of these variables.

During childbirth, more complications occurred in the preterm group (41.7% versus 33.3%); however, it showed no statistical significance. The most common type of delivery was cesarean section (86.4% versus 85.6%) in both groups but without relevant differences. Most surgical deliveries were due to maternal or fetal indications.

Significant differences were found regarding the presentation at delivery and in terms of birth weight in relation to GA, considering that preterm infants had more frequent pelvic or cormic presentation (14.4% versus 6.3%) and low birth weight for GA (32.2% versus 18.3%). The male sex was predominant (56.9%), but no differences were observed between the two newborn groups.

The variables of each block, which were significant in bivariate analysis, were submitted to a new multivariate analysis (Table 2). The variables that maintained significant association with prematurity in the final Poisson regression model after adjusting for the other factors were greater number of prenatal consultations (RR = 0.924; 95%CI: 0.901–0.947), which showed to be protective factor, being a primiparous mother (1.104 95%CI: 1.004–1.213), and having hypertensive syndromes of pregnancy (RR = 1.262; 95%CI: 1.161–1.371), which proved to be risk factors.

Significant risk factors identified in the bivariate analysis (thrombophilia, bleeding in the third trimester of pregnancy) and in the multivariate block analysis (pelvic or cormic presentation at delivery and newborn small for GA) were not confirmed in the final model. No sociodemographic, environmental, and newborn characteristics remained factors associated with prematurity in the final model.

## 4. Discussion

The frequency of prematurity in NICU among those eligible was 76.9% (the frequency of eligible premature births in relation to the total number of births in this period was 3.8%). The prevalence of prematurity among live births between 2013 and 2018 at this hospital was 23%. Most premature newborns had a GA of fewer than 34 weeks. This result was higher than that recorded by DATASUS in the same period in Fortaleza, in the state of Ceará and in Brazil (11.4%, 11.8% and 27.8%, respectively) [7]. Another study from Manaus-Amazonas, in the northern region of Brazil, showed a prevalence of prematurity of 10% [41]. In Colombia, another study [42] also reported that the prevalence of premature births represented 11% of births. The frequency reported in our study was high, which can be explained by the fact that the institution where the births took place is considered a referral hospital for high-risk pregnancies and by the fact that the sample was restricted to those admitted to the NICU. Similar findings were demonstrated in the study by Bezerra et al. [43], with a higher prevalence of preterm births at 66.3%.

In the current study, the parturients were mostly young adults with higher education (complete or incomplete) and married or in a common-law marriage, but none of the parturients’ sociodemographic and environmental characteristics were associated with prematurity. Unlike other studies [12,22,23,24,25,26,27,28], several sociodemographic, demographic, genetic, and health factors had a statistically significant association with preterm birth. For Sampaio et al. [23], the level of education directly influences the quality of care the pregnant woman adopts since the moment of conception.

Regarding prenatal consultations, we found that 13.5% of the parturients had fewer than five prenatal consultations. Even though the parturients had varying economic levels, they had a health plan that gave them access to quality prenatal care. Different results were found in the study by Almeida et al. [12], who found a high frequency of mothers of preterm infants with inadequate prenatal care (with fewer than five consultations). In the study conducted by Vanin et al. [39], incomplete prenatal care was a predictor of late preterm birth, which is consistent with what has been described by other authors [19,23,26,43]. It should be noted that fewer prenatal consultations are expected in pregnancies terminated early when compared to full-term pregnancies.

In this investigation, as the group of postpartum women with full-term newborns was small (*n* = 4), we chose to use the variable in the continuous form in the bivariate and multivariate analysis models. With an increase in the number of prenatal consultations, a significant protective effect was observed for the occurrence of prematurity in the final model (RR = 0.924; 95% CI: 0.901–0.947).

In this study, a substantial portion of the data was obtained from clinical records, and there may be other unassessed factors affecting the occurrence of preterm births. The sample size may not have allowed for the clarification of the role of some factors whose effect is smaller. This study enabled the mapping of the environments of parturients residing in Fortaleza with regard to hydrosanitary conditions. This study showed that more than 98% of the parturients had access to clean water, but only 51.5% of them had a sewer system installed in their homes. These findings were not associated with the occurrence of preterm births. Ramos and Cumon [44] showed that social, economic, and sanitary conditions of the environment where the children were conceived and born were associated with the preterm infants’ future quality of life.

The literature shows an increase in the number of women who become pregnant aged 34 years and over [22,25,27]. Chermont et al. [25] reported an increase in preterm births, with an increase in the number of primiparous women aged over 34 years. These results are different from those of the current study, which showed an increase in the group aged 35 years or less, corresponding to 78.1% of the 480 parturients, but without significant association with prematurity. 

On the other hand, extremes of mother age (under 20 years and older) showed a higher prevalence of prematurity, corroborating data from the literature [45,46,47,48].

Several studies have associated the presence of maternal comorbidities as independent risk factors for preterm births, such as hypertensive syndrome and previous or gestational diabetes mellitus [2,21,24,30]. In the present study, hypertensive syndrome stood out among the risk factors for prematurity and remained in the final model (RR = 1.262; 95%CI: 1.161–1.371). This result is corroborated by findings of other publications [12,13,24,26,30]. The frequency of gestational diabetes mellitus (7.3% in mothers of preterm infants versus 9.9% of full-term infants) did not reach statistical significance. This is dissimilar from Brandi et al. [30], where the presence of previous or gestational diabetes mellitus was identified as a significant risk factor for prematurity. A cross-sectional study conducted in Northeast Brazil identified primiparity as a significant risk factor for preterm birth (*p* = 0.044) [12]. In another investigation, no effect of primiparity was identified [30], similar to the present study, in which primiparity lost its statistical significance in the multivariate analysis by block. In the present study, 57.5% of the mothers of preterm infants and 44.1% of those of full-term infants were primigravidae. The first pregnancy was shown to be a significant risk factor (RR = 1.104; 95% CI: 1.004–1.213) for prematurity in the final model of the multivariate analysis, corroborating the results published by Bezerra et al. [41]. A systematic review of 41 studies found that nulliparity is associated with a significantly higher risk of low birth weight or small for gestational age births; there may be confounding factors that could influence the association found. High multiparity, although associated with reduced birth weight, was not associated with low birth weight or preterm birth [49].

Various etiologies are associated with vaginal bleeding and require the termination of pregnancy before 37 weeks, such as placenta previa, placenta accreta, vasa previa, and fetal growth restriction. The rate of placenta accreta is increasing, which is why monitoring vaginal bleeding with frequent uterine contractions and hemoglobin levels is important, as these may be factors suggesting emergency cesarean surgery [50]. In our study, 6.3% experienced hemorrhage in the third trimester of pregnancy, but no significant differences were found when comparing preterm and term newborns.

A higher frequency of prematurity (32.2%) was observed in newborns with low weight for GA than in full-term ones (18.3%), corroborating the results of the study by Brandi et al. [30].

In bivariate analysis by block, significant risk factors for prematurity were pelvic or cormic presentation at delivery (RR = 1.162; 95% CI: 1.051–1.284) and low birth weight for GA (RR = 1.147; 95% CI: 1.047–1.256), but this effect disappeared in the multivariate analysis final model. Therefore, the newborn’s characteristics were not associated with prematurity, which is not observed in other studies [19,30].

In the final model, the only variables associated with an increased risk of prematurity were being a primiparous or nulliparous mother and having hypertensive syndromes during pregnancy, whereas the increase in the number of prenatal consultations was a protective factor. These results are like those by Chermont et al. [25].

Prematurity is a multifactorial syndrome, determined by different situations and comorbidities, with several associated risk factors; the present study is expected to contribute as a first step in the great challenge of preventing it.

The limitations of this study include the fact that data from the clinical records of parturients and newborns were used, and there may have been variables that were not assessed that could be associated with preterm birth, as well as the lack of recording of any relevant data (missing values); the inclusion of parturients living in the municipality of Fortaleza was also aimed at studying environmental factors, such as the water and sanitation conditions of homes in Fortaleza, which led to a reduction in the sample size compared with the number of admissions to the NICU and which may not have made it possible to better clarify the role of some factors whose effect was smaller.

As for this study’s positive aspects, we have the mapping of parturient women’s homes in terms of water and sanitation conditions, showing a significant percentage of homes that had access to treated water (98%) but that only 51.5% had a sewage system connected to their homes. Prematurity can and should be avoided as long as the patient is properly monitored, controlling blood pressure levels throughout pregnancy, and avoiding serious conditions such as severe pre-eclampsia, eclampsia, and HELLP syndrome, which lead to premature birth. Furthermore, primiparous women were significantly associated with prematurity, showing that today, it is increasingly possible to get pregnant with assisted reproduction technology. Even though the sociodemographic factors of the parturients were not statistically significant, the literature shows that they are relevant. The fact that primigravida is a determining factor in prematurity shows managers the importance of adequate prenatal care.

## 5. Conclusions

Significant positive associations were found between preterm birth and the presence of hypertensive syndromes during pregnancy and being a primiparous woman, whereas there was a negative association with an increase in the number of prenatal consultations, so special attention should be given to primiparous pregnant women and their complications.

This study contributed to providing greater visibility to prenatal care and understanding the complications of pregnancy and childbirth care. It emphasized the importance of prenatal care in identifying pregnant women with a greater possibility of preterm birth, ensuring follow-up, and adopting appropriate preventive measures to reduce prematurity and, consequently, decrease neonatal and postneonatal mortality.

## Figures and Tables

**Table 1:** Sample description according to maternal, environmental, and newborn characteristics. Bivariate association analysis and relative risk estimate for the prematurity outcome in a cohort of 480 parturients and their respective newborns in the city of Fortaleza-CE, Brazil.

VARIABLES (*n* = 480)	All	Gestational Age		Bivariate		
		Preterm (<37 Weeks)	Full-Term NB ^1^ (37 to 41 Weeks and 6 Days)			
				*p*-Value *	RR	95%CI
Distribution by Block	*n*(%)	*n*(%)	*n*(%)			
1. Maternal sociodemographic characteristics and environmental factors						
Age (years) ^2^	31 ± 5.7; 13–58	31 ± 5.6	32 ± 6.0	0.405	0.996	0.987–1.005
>35	105(21.9)	78(21.1)	27(24.3)	0.513	1.000	-
≤35	375(78.1)	291(7.9)	84(75.7)		1.045	0.922–1.184
Marital status						
Single/No partner	117(24.4)	85(23.0)	32(28.8)	0.210	1.000	-
Married/With partner	363(75.6)	284(77.0)	79(71.2)		1.077	0.952–1.219
Level of education						
Elementary I/Elementary II	19(4.0)	14(3.8)	5(4.5)	0.327	0.973	0.737–1.285
High school	148(30.8)	112(30.4)	36(32.4)		0.999	0.890–1.122
Incomplete higher education	70(14.6)	59(16.0)	11(9.9)		1.113	0.984–1.260
Complete higher education	243(50.6)	184(49.9)	59(53.2)		1.000	-
Available sewage system						
Yes	247(51.5)	177(48.0)	56(50.5)	0.666	0.977	0.886–1.078
No	233(48.5)	192(52.0)	55(49.5)		1.000	-
Potable water						
Yes	471(98.1)	5(1.4)	4(3.6)	0.222	0.719	0.4–1.292
No	9(1.9)	364(98.6)	107(96.4)		1.000	-
2. Pregnancy and childbirth conditions and maternal complications						
Number of prenatal consultations ^2^	7 ± 2.3; 1–15	6 ± 2.2	8 ± 2.2	<0.001	0.922	0.899–0.946
<5	65(13.5)	61(16.5)	4(3.6)			
≥5	415(86.5)	308(83.5)	107(96.4)			
Number of pregnancies ^2^	1.8 ± 1.1; 1–8	1.7 ± 1.1	1.9 ± 1.0	0.266	0.973	0.928–1.021
1	261(54.4)	212(57.5)	49(44.1)	0.017	1.133	1.024–1.254
≥2	219(45.6)	157(42.5)	62(55.9)		1.000	-
History of miscarriage						
Yes	106(22.1)	78(21.1)	28(25.2)	0.363	0.946	0.834–1.073
No	374(77.9)	291(78.9)	83(74.8)		1.000	-
Hypertensive syndrome during pregnancy ^3^						
Yes	124(25.8)	113(30.6)	11(9.9)	<0.001	1.267	1.164–1.38
No	356(74.2)	256(69.4)	100(90.1)		1.000	-
Gestational diabetes						
Yes	38(7.9)	27(7.3)	11(9.9)	0.422	0.918	0.745–1.132
No	442(92.1)	342(92.7)	100(90.1)		1.000	-
Urinary tract infection						
Yes	86(17.9)	69(18.7)	17(15.3)	0.481	1.054	0.936–1.186
No	394(82.1)	300(81.3)	94(84.7)		1.000	-
TORCHS ^4^						
Present	13(2.7)	9(2.4)	4(3.6)	0.509	0.898	0.623–1.295
Absent	467(97.3)	360(97.6)	107(96.4)		1.000	-
Thrombophilia						
Yes	14(2.9)	13(3.5)	1(0.9)	0.206	1.215	1.042–1.418
No	466(97.1)	356(96.5)	110(99.1)		1.000	-
Bleeding in the 3rd trimester of pregnancy						
Yes	30(6.3)	27(7.3)	3(2.7)	0.114	1.184	1.040–1.349
No	450(93.8)	342(92.7)	108(97.3)		1.000	-
Presence of complications during labor						
Yes	191(39.8)	154(41.7)	37(33.3)	0.122	1.084	0.984–1.194
No	289(60.2)	215(58.3)	74(66.7)		1.000	-
Delivery type						
Cesarean section	414(86.3)	319(86.4)	95(85.6)	0.875	0.017	0.879–1.177
Vaginal delivery	66(13.8)	50(13.6)	16(14.4)		1.000	-
3. Characteristics of the fetus						
Presentation at birth						
Cephalic	420(87.5)	316(85.6)	104(93.7)	0.022	1.000	-
Pelvic or cormic	60(12.5)	53(14.4)	7(6.3)		1.174	1.055–1.307
Sex						
Male	273(56.9)	208(56.4)	65(58.6)	0.743	0.914	0.595–1.406
Female	207(43.1)	161(43.6)	46(41.4)		1.000	-
Birth weight for gestational age according to the classification of WHO ^5^ (*n* = 473) ^9^						
SGA ^6^	138(29.2)	119(32.2)	19(18.3)	0.005	2.129	1.237–3.666
AGA ^7^ or LGA ^8^	335(70.8)	250(67.8)	85(81.7)		1.000	-

^1^NB: newborns; ^2^ Variable described as mean ± standard deviation; minimum value—maximum value; ^3^ Hypertensive syndrome during pregnancy includes mild pre-eclampsia, severe pre-eclampsia, eclampsia and HELLP syndrome; ^4^ TORCHS: toxoplasmosis, rubella, cytomegalovirus, herpes simplex, and syphilis; ^5^ WHO: World Health Organization; ^6^ SGA: small for gestational age; ^7^ AGA: adequate for gestational age; ^8^ LGA: large for gestational age; ^9^ measurements that can be obtained pre-birth were obtained and proven at birth (weight was substituted by the birth weight for gestational age according to the WHO classification). *p*-value *: Fisher’s test or Pearson’s chi-square test (categorical variables) and Mann–Whitney test (quantitative variables).

**Table 2:** Multivariate association analysis and relative risk estimate for the prematurity outcome in a cohort of 480 parturients and their respective newborns in the city of Fortaleza-CE, Brazil.

VARIABLES (*n* = 480)	Multivariate Analysis *					
	Per Block			Final		
Distribution by Block	*p*-Value	RR	95%CI	*p*-Value	RR	95%CI
1. Maternal sociodemographic characteristics and environmental factors						
2. Conditions of pregnancy and childbirth and maternal complications						
Number of prenatal consultations ^1^	<0.001	0.924	0.901–0.947	<0.001	0.924	0.901–0.947
Number of pregnancies						
1	0.040	1.104	1.004–1.213	0.040	1.104	1.004–1.213
≥2		1			1.000	
Hypertensive syndrome during pregnancy ^2^						
Yes	<0.001	1.262	1.161–1.371	<0.001	1.262	1.161–1.371
No		1			1.000	
3. Characteristics of the fetus						
Presentation at birth ^7^						
Cephalic	0.003	1	-	-	-	-
Pelvic or cormic		1.162	1.051–1.284	-	-	-
Birth weight for gestational age according to the classification of WHO ^3^ (*n* = 473) ^7^						
SGA ^4^	0.003	1.147	1.047–1.256	-	-	-
AGA ^5^ or LGA ^6^		1	-	-	-	-

^1^ Variable described quantitatively, by increment of one unit; ^2^ Hypertensive syndrome during pregnancy includes mild pre-eclampsia, severe pre-eclampsia, eclampsia, and HELLP syndrome; ^3^ WHO: World Health Organization; ^4^ SGA: small for gestational age; ^5^ AGA: adequate for gestational age; ^6^ LGA: large for gestational age; ^7^ measurements that can be obtained pre-birth were obtained and proven at birth (weight was substituted by the birth weight for gestational age according to the WHO classification). * Multivariate: Poisson regression with robust variation and Wald statistic were used.

## Data Availability

The data presented in this study are available on reasonable request from the corresponding author, although raw data will not be shared due to privacy restrictions of the participants.
